# Stay-at-home orders and the willingness to stay home during the COVID-19 pandemic: A stated-preference discrete choice experiment

**DOI:** 10.1371/journal.pone.0253910

**Published:** 2021-07-01

**Authors:** Liqing Li, Dede Long, Mani Rouhi Rad, Matthew R. Sloggy

**Affiliations:** 1 Department of Economics, California State University Fullerton, Fullerton, CA, United States of America; 2 Department of Economics, California State University Long Beach, Long Beach, CA, United States of America; 3 Agricultural Sciences, Clemson University, Clemson, SC, United States of America; 4 Department of Agricultural and Resource Economics, Colorado State University, Fort Collins, CA, United States of America; University of Florida, UNITED STATES

## Abstract

The spread of COVID-19 in the Spring of 2020 prompted state and local governments to implement a variety of policies, including stay-at-home (SAH) orders and mandatory mask requirements, aimed at reducing the infection rate and the severity of the pandemic’s impact. We implement a discrete choice experiment survey in three major U.S. States—California, Georgia, and Illinois—to empirically quantify individuals’ willingness to stay (WTS) home, measured as the number of weeks of a potential new SAH order, to prevent the spread of the COVID-19 disease and explore factors leading to their heterogeneous WTS. Our results demonstrate broad support for statewide mask mandates. In addition, the estimate of WTS to lower new positive cases is quite large, approximately five and half weeks, even though staying home lowers utility. We also find that individuals recognize the trade-offs between case reduction and economic slowdown stemming from SAH orders when they decide to stay home or not. Finally, pandemic related factors such as age, ability to work from home, and unemployment status are the main drivers of the heterogeneity in individuals’ WTS.

## Introduction

The spread of COVID-19 across the United States in the Spring of 2020 prompted state and local governments to implement a variety of policies, including stay-at-home (SAH) orders and mandatory mask requirements, aimed at reducing the infection rate and the severity of the pandemic’s impacts. As the virus continued to spread, several state governments re-implemented SAH orders. For example, Oregon imposed a one-week “freeze” under which businesses were required to close their offices and mandate work-from-home starting November 18, 2020. New Mexico also re-implemented a two-week SAH order that was in effect from November 16 through November 30, 2020. While such policies lower the transmission rate [1–3], reduced economic activity and social interactions due to the lockdown have also resulted in economic and psychological costs [4–7].

Existing literature in economics has extensively examined the costs and benefits of national-level lockdowns applying modified Susceptible-Infectious-Recovered models to incorporate varying individual behaviors and demographic compositions [1, 8–15]. These studies have focused on the trade-offs from the policy-makers’ perspective and shown that lockdowns, especially those that differentially target older and riskier groups, can generate benefits that exceed program costs. However, these studies impose assumptions on individuals’ willingness to stay (WTS) at home. Only a few studies have examined how the general public values the costs and benefits of these policies [16, 17]. To better understand individuals’ willingness to comply with SAH orders, we need to examine their preferences regarding the length of potential lockdowns. Unfortunately, such preferences are difficult to measure using reveal-preference information because the impacts of SAH orders are often correlated with their lengths. As a result, we need a hypothetical experiment that allows us to vary attributes of a SAH order independent of each other to measure individuals’ WTS.

In this paper, we examine individuals’ WTS home to prevent the spread of the COVID-19 disease and factors driving their heterogeneous WTS. We implement a discrete choice experiment (DCE) survey to quantify respondents’ WTS depending on five attributes: the length of a new SAH order, state-level increases in the number of newly confirmed cases, increases in the number of unemployment insurance claims, the probability of schools opening, and whether a mask wearing mandate is implemented. Individuals’ decisions of staying home have a direct impact on both the reduced new positive cases and the negative psychological and economic impact. These positive and negative outcomes are also correlated with each other. Consequently, it is difficult to disentangle the effect of these variables on individuals’ utility and to evaluate the potential tradeoff. Implementing a DCE allows us to construct hypothetical scenarios by varying different attributes, delineating the tradeoffs that are vital to individuals’ decision-making and providing flexibility to measure respondents’ WTS. Our survey targets three states in the U.S.: California, Georgia, and Illinois. These three states are drastically different in the number of infections and deaths per capita, the timing of surges in positive cases, and most importantly, policy responses towards the COVID-19 pandemic including the implementation of the SAH order, schools opening, and mask wearing mandate.

### Survey design

**Fig 1:**
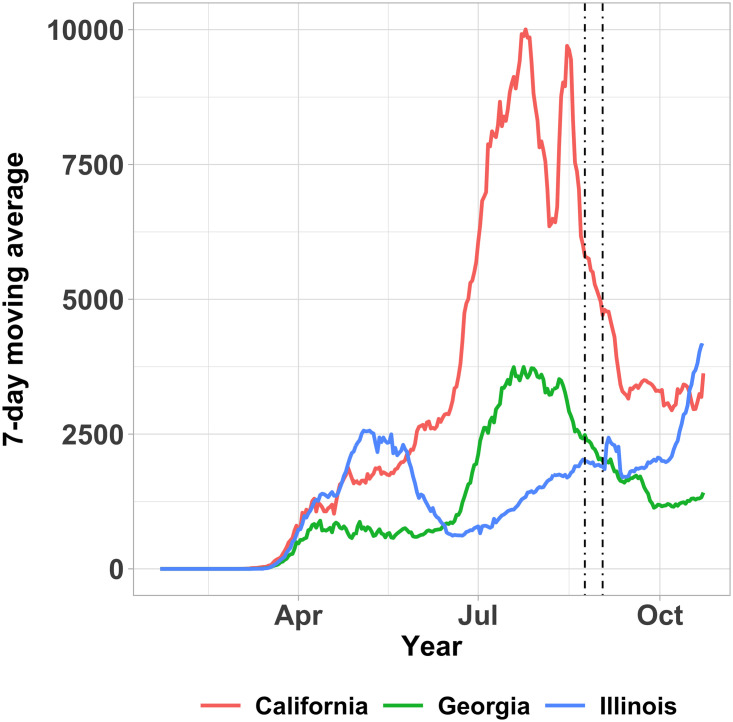
7-day moving average of the number of cases across the three states. Dashed lines show the beginning and end of the survey period. Data source: https://covid.cdc.gov/covid-data-tracker.

We conduct a stated-preference DCE survey to estimate residents’ WTS home during the COVID-19 pandemic in California, Georgia, and Illinois. The survey was implemented during the final week of August, 2020. All three states have gradually lifted SAH mandates between the end of April and end of May. At the time of the survey, California had experienced its deadliest month since the pandemic started with the highest number of new cases in the nation [18, 19]. At the same time, the number of newly confirmed cases was relatively stable in Illinois, while Georgia’s number of infections had slowly tapered as shown in Fig 1.

The DCE method provides us with the flexibility needed to estimate marginal values for each attribute included in the survey design [20]. Our survey was administered electronically through the online survey platform Qualtrics, and it took respondents an average of 20 minutes to finish. Web-based survey tools like Qualtrics provide flexibility to randomize question order and cost-effectively select targeted populations. We acknowledge that volunteer survey takers might have a higher than average willingness to pay (WTP) for market or non-market goods, but it is unclear whether the selection bias has a similar effect on WTS that is evaluated in our study.

**Fig 2:**
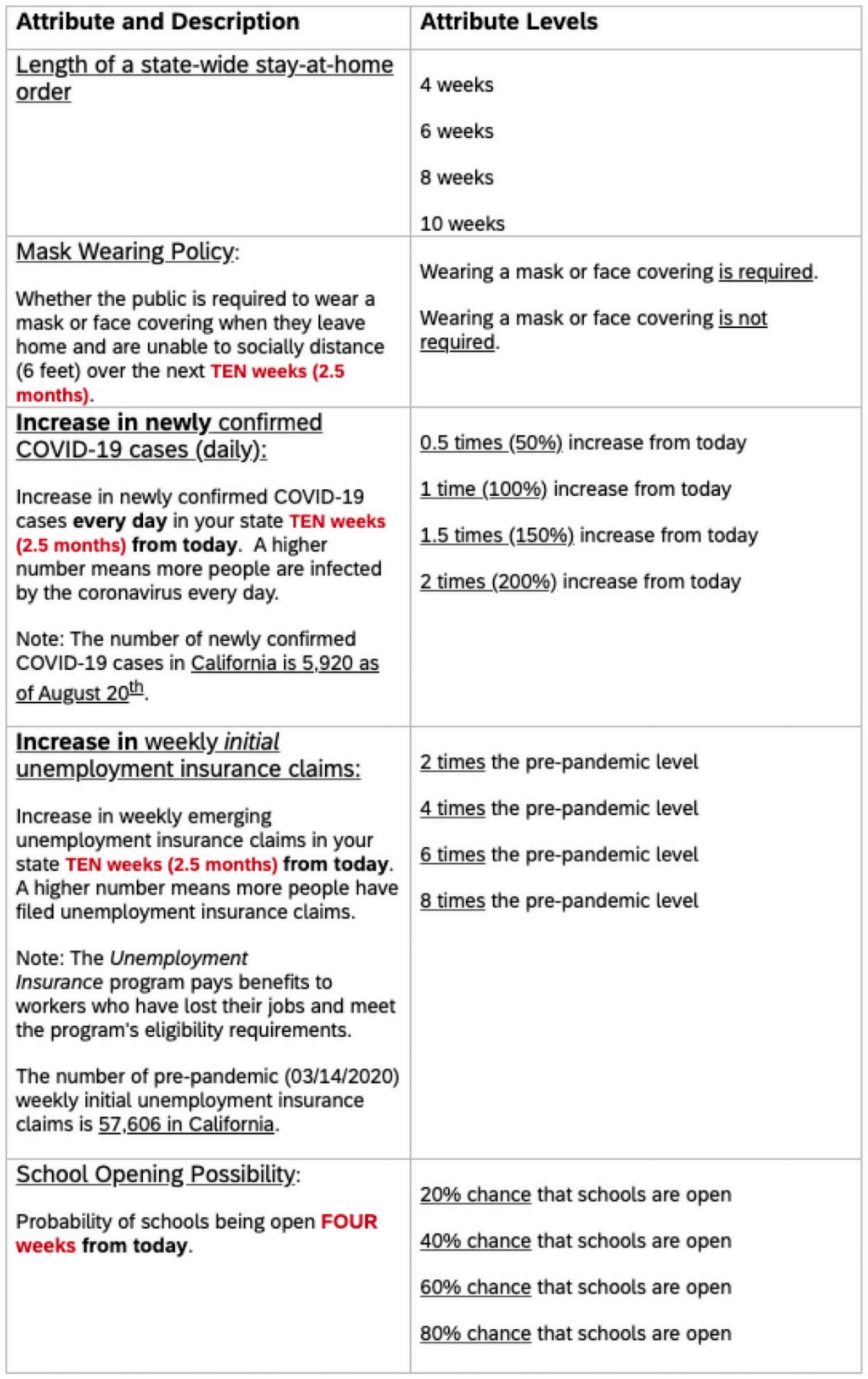
Attribute levels in the discrete choice experiment survey.

We first have the respondents read important background information on the COVID-19 pandemic to contextualize them with the issue. Specifically, we include information on effective preventative measures (i.e., social distancing), public policy that has been implemented (i.e., SAH orders), the positive and negative consequences of this policy (i.e., case reduction and economic and psychological cost), and the possibility of a new round of SAH orders given the new outbreaks in many states after the reopening. We then present respondents a description of the status quo scenario without a SAH order when states are partially open. Next, we show the fixed and variable attributes if a new SAH order is implemented. The fixed features include what activities and businesses are open and closed under a SAH order. The variable features of SAH scenarios include the length of SAH order, mask wearing policy, increase in newly confirmed COVID-19 cases (daily), increase in weekly initial unemployment insurance claims, and the probability of schools opening. Descriptions and levels of variable attributes used in the survey is given in Fig 2.

**Fig 3:**
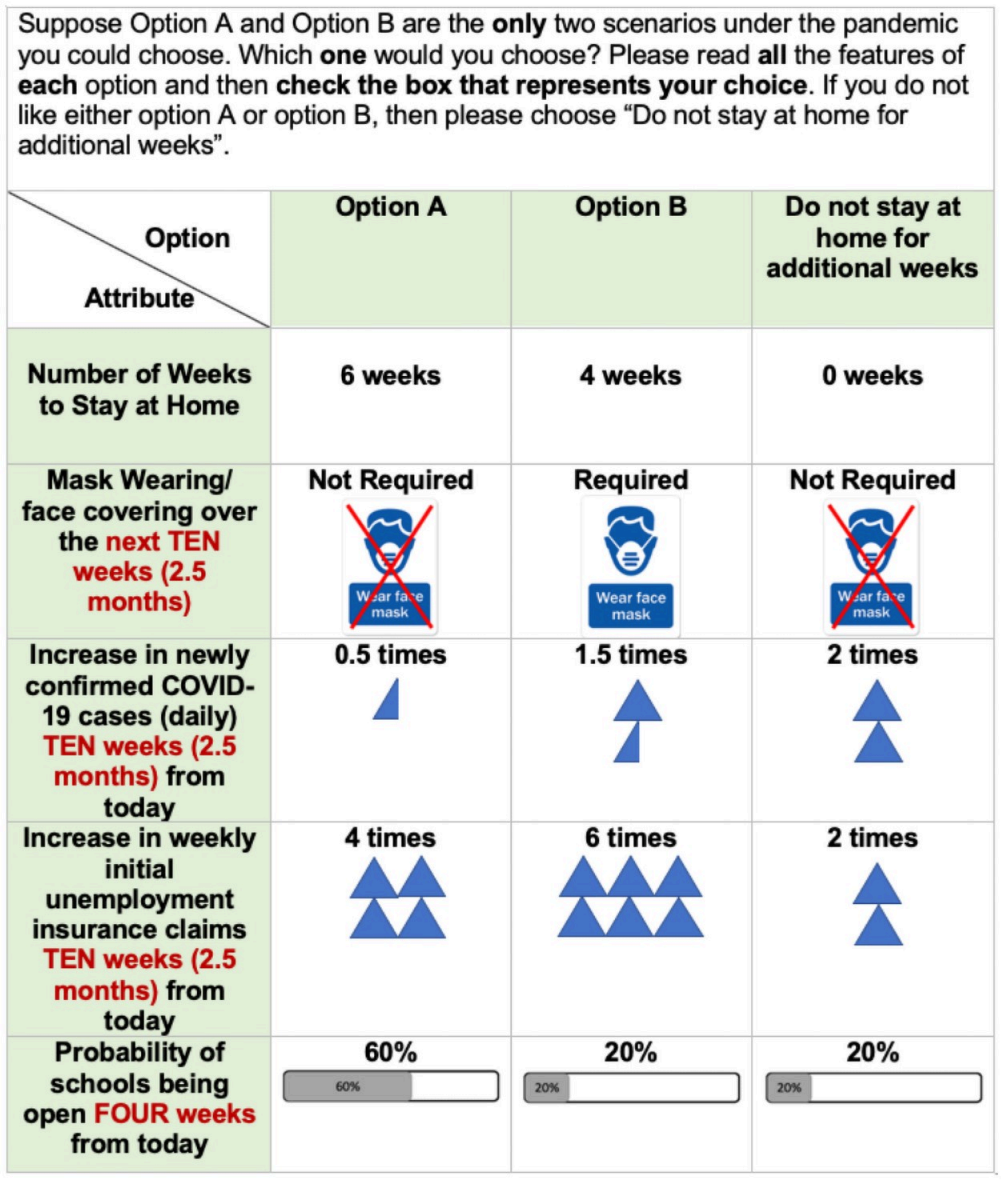
Sample choice card.

The background and fixed and variable attributes information are followed by a total of six discrete-choice questions. Within each of the six discrete-choice questions, we ask respondents to choose between a pair of hypothetical SAH scenarios with different variable attribute levels and a status quo scenario (i.e. No new SAH policy is implemented and the state remains partially reopen). A sample choice card is available in Fig 3. After the discrete-choice questions, we ask respondents for information on their demographics. Some pandemic related information (e.g. whether respondents are health or essential workers, if they have family members who are health workers, and if respondents can work from home) are also surveyed.

#### Attribute and attribute levels

To elicit individuals’ WTS, we develop a set of realistic SAH scenarios based on available data, including a status quo scenario when respondents choose not to have a new state-level SAH policy. The length of a SAH order is the first attribute in each choice question—0 weeks, 4 weeks, 6 weeks, 8 weeks, or 10 weeks, which serves as the non-monetary payment vehicle similar to the monetary cost in a traditional WTP study. Levels of the other choice attributes vary depending on the lengths of the SAH order. Zero weeks (no SAH order) is the baseline and is presented as the status quo option in each choice question. We choose ten weeks (2.5 months) as the maximum SAH order lengths given that the medical community is pushing forward with the COVID-19 vaccine development, and studies have shown it is likely to be available 12–18 months from the start of the pandemic [21, 22] and based on the length of existing policies [23].

The second attribute, a binary variable indicating whether there is a mask mandate or not, indicates whether the government imposes a statewide mask wearing mandate so that respondents are required to wear masks or face coverings when they leave home or are unable to social distance (i.e., stay 6-feet apart) over the next ten weeks (2.5 months) from the day the survey was taken. The third attribute is the increase in daily newly confirmed COVID-19 cases, a continuous variable. The attribute levels are presented as the percentage increase in the next ten weeks from the day the respondents answer the survey—0.5 times (50%), 1 time (100%), 1.5 times (150%), and 2 times (200%). In the attribute description, we provide respondents the number of newly confirmed cases in their state on August 20, which was ten days before the survey was taken, as a reference to help them quantify the attribute levels. This attribute captures the benefits, “low increase” (reduction) of newly positive COVID-19 cases, from staying home.

We also introduce the increase in weekly initial unemployment insurance claims as continuous variable—2 times, 4 times, 6 times, and 8 times the pre-pandemic level ten weeks from the day the survey was taken, as the fourth attribute. Similarly, in the attribute description, we present respondents with the number of pre-pandemic (as of March 14, 2020) weekly initial unemployment insurance claims in their corresponding states.

The final attribute is the probability of schools opening four weeks from the date when the survey was taken—either a 20%, 40%, 60%, or a 80% probability. Some schools in the USA have started in-person instruction at the time the survey was distributed and already observed outbreaks of cases among students [24]. While some schools continued to conduct in-person teaching, others have switched to a virtual learning environment in response to the spike in infections. Whether schools remain open depends on whether individuals are willing to stay home and practice social distancing. Considering the great uncertainty around the timing of school opening and to ensure the validity of our survey, we choose to use “four weeks” from the date when the survey was taken instead of ten weeks as the time frame when describing the school opening choice attribute. We acknowledge that even though this makes our survey design more realistic, it also creates potential inconsistency by comparing an attribute at the end of a SAH order to another attribute midway through the SAH order given the time frame in other choice attributes is ten weeks. Overall, for all attributes except the mask mandate attribute, we use percentages relative to a baseline instead of absolute numbers to help respondents comprehend and compare the attribute levels since it may be difficult to contextualize and internalize differences between numbers of different orders of magnitude.

#### Pilot survey

Various survey drafts were shown to faculty members, graduate and undergraduate students, survey design specialists, and other members of the general public to solicit their opinions of the survey. The feedback from the test survey was used to make numerous changes in the survey, particularly to improve the clarity of the information presented on the background information and the descriptions of the choice attributes and the layout of the choice questions. We also adjusted the number of questions and the graphical presentations of attribute levels. Due to the fast development of the pandemic and the consequent time constraint, we were not able to conduct formal focus groups to test the survey. However, the feedback we solicited from a diverse group helped us improve the survey substantially.

A pilot version of the survey was sent out in mid-August through Qualtrics. After the pilot survey pre-launch, we obtained 51 complete and usable surveys. The pilot survey results allowed us to: 1) evaluate whether respondents are responsive to the lengths of the SAH order. For example, whether extremely long SAH periods deter respondents from choosing to stay home. 2) modify any problems or issues with the survey; 3) evaluate whether respondents understand the survey questions. We refined the final survey based on the pilot study results by increasing the lengths of stay from 0, 2, 4, 6, 8 weeks to 0, 4, 6, 8, 10 weeks. In addition, we reminded the respondents multiple times in the survey that the SAH order is strict, implying that individuals’ daily activities will be severely restricted under the mandate.

#### Survey validity

To ensure the validity and reliability of the value estimates in a DCE study, survey questions need to be consequential. In particular, participants must perceive that their responses can potentially affect policy implementation [25]. We first include a policy consequentiality script in the introduction to convince respondents that their answers can possibly affect COVID-19 public policy implementation. In addition, choice questions are followed by a consequentiality question asking respondents to what extent they believe their answers will be considered by policymakers.

Following the standard practice in DCE, we use a *D*_0_-optimal design [26] with multiple restrictions to allocate attribute levels to non status-quo options in choice questions. For example, long SAH lengths are more likely to lead to a reduction in newly confirmed COVID-19 cases, a high increase in weekly unemployment insurance claims, and a high probability of schools opening. Our experimental design ensures that no options dominate or will be dominated within or across choice questions. The final design consists of a total of 18 choice questions with three blocks of six questions in each block. Both the order of choice questions and blocks are randomized to minimize bias due to learning from earlier questions and survey fatigue. We also prevent respondents from going back and change their answers to previous questions. An example of the full survey is presented in the online S1 section in S1 File. Full Survey.

### Data

We collected a total of 731 complete and usable responses. We only include individuals who choose “I will read carefully and provide my best answers” in the consent questions. This resulted in us dropping 71 responses. To ensure within state variation and representation of both urban and rural areas, we weighted the number of surveys collected by the share of the state population residing in urban and rural counties. In the end, we have a total of 479 responses from urban counties, and 192 responses from rural counties.

To investigate the demographics of our respondents further, we present the summary statistics of our survey sample in Table 1, columns 4 through 6. Alongside these, we also report the actual demographics of each state, derived from the American Community Survey (ACS) obtained from the Census Bureau, in columns 1 through 3. Table 1 shows that the respondents have a higher proportion of whites, with lower proportions of minorities. They are also more likely to be female, younger, and from lower income brackets. Stars in the table indicate whether the differences between the population and our sample are statistically different from zero, obtained from one-sample proportional tests. The test results show that the majority of demographics in our survey are different from the population. Past research has demonstrated that less-educated, lower-income, and older (age 65 and older) individuals are generally underrepresented in internet surveys due to the lack of access to the internet [27]. Depending on the direction of correlation between individual demographics and their WTS, our estimates are likely to have an upward or a downward bias.

**Table 1:** Distribution of demographic characteristics of survey participants and the 2019 state-level population.

Category	Variable	Population			Survey Sample		
		(1) California	(2) Georgia	(3) Illinois	(4) California	(5) Georgia	(6) Illinois
Age	18–24	9.30	9.70	9.20	11.67***	9.02**	15.16***
	25–34	15.30	13.80	13.80	25.83***	21.72***	20.49***
	35–44	13.40	13.20	13.00	22.92***	23.36***	24.18***
	45–54	12.60	13.10	12.60	13.33	17.62***	14.75***
	55–64	12.10	12.30	13.00	12.92***	17.21***	14.34***
	65+	14.80	14.40	16.20	13.33***	11.07***	11.07***
Gender	Male	49.71	48.69	49.06	46.25***	33.2***	34.84***
Race/Ethnicity	White	63.60	59.90	73.80	62.50	71.72***	79.51***
	Black or African American	7.00	33.50	15.40	4.58***	22.13***	9.84***
	American Indian and Alaska Native	2.00	0.90	0.80	1.67	0.41***	0.41***
	Asian	17.10	4.90	6.60	12.08***	1.64***	3.28***
	Native Hawaiian and Other Pacific Islander	0.80	0.20	0.10	0.42***	0.00	0.00
	Hispanic	39.40	9.80	17.50	17.08***	3.28***	6.97
	Other	14.94	3.50	6.30	1.67	0.82	0.00
Marital Status	Single	37.35	34.74	35.74	40.83	35.25	38.93
	Married	46.54	46.24	47.05	47.92	47.13	42.62
	Divorced	9.28	11.34	9.89	7.92	12.7	13.11
	Widow(er)	4.93	5.44	5.74	3.33	4.92	5.33
Education Level	Less than high school graduate	15.97	12.09	10.15	2.08	4.92	1.64
	High school graduate or GED	20.59	27.40	25.94	15.42	22.95	20.49
	Some college or associate’s degree	28.44	28.00	28.15	32.92	34.01***	41.80***
	Bachelor’s degree	21.86	19.92	21.69	31.25***	22.13***	20.90
	Graduate or professional degree	13.14	12.59	14.06	18.33	15.98	15.16
Income Level	Less than $25,000	14.90	19.30	17.60	16.25*	25.82***	19.67***
	$25,000 to $49,999	16.70	21.30	19.40	21.25***	22.13	25.00***
	$50,000 to $74,999	15.30	18.30	16.50	19.58***	21.72***	21.72***
	$75,000 to $99,999	12.50	12.70	12.80	13.33	11.48***	14.75
	$100,000 to $149,999	17.40	14.80	16.90	14.17	9.84	10.25
	$150,000 to $199,999	9.40	6.30	7.80	7.50	5.33	3.69
	$200,000 or more	13.70	7.30	9.00	7.92***	3.69***	4.92***

### Methods

Our survey design was examined by Clemson University and California State University Institutional Review Board in 2020, and received a notice of exemption (Clemson University Proposal number 2020–160; California State University Proposal number 21–008). There was no deception used in the survey design. Participants have provided written consent for their responses to be used in this study at the beginning of the survey.

We examine individuals’ preferences towards a SAH policy using the DCE method. Our empirical model is based on the discrete choice random-utility maximization (RUM) framework discussed in [28]. Individuals choose from a set of SAH scenarios with varying attributes to maximize their utility. The utility of individual *n* choosing option *i* in choice scenario *t* can be written as:

$$\begin{array}{c} U_{nit}=\beta_{\text{n}}^{\prime }{\text{X}}_{\text{nit}}+\epsilon_{nit} \end{array}$$ (1)

**X**_**nit**_ here is an observed component, which is a vector of alternative-specific attributes including the number of daily newly confirmed cases, number of weekly unemployment insurance claims, statewide mask mandate, probability of schools opening, and non-monetary cost measured as the length of a strict SAH mandate. *β*_*n*_ is a vector of individual-specific random coefficients. *ϵ*_*nit*_ is an unobserved random component that captures individual’s idiosyncratic tastes and is i.i.d extreme value type-one distributed [29].

The chosen alternative can be specified as *y*_*n*_. Conditional on *β*_*n*_, the probability of individual n choosing alternative i over t scenarios where i ≠ j is:

$$\begin{array}{c} L\left(y_{n}|\beta_{n}\right)=\prod_{t}\frac{e^{\beta_{ni}x_{it}}}{\sum_{j}e^{\beta_{nj}x_{jt}}} \end{array}$$ (2)

Since *β*_*n*_ is a random coefficient, integrating over all possibilities of *β*_*n*_ gives us the unconditional probability of choosing alternative j for individual n as:

$$\begin{array}{c} P_{n}\left(y_{n}|\theta \right)=\int L\left(y_{n}|\beta_{n}\right)f\left(\beta |\theta \right)d\beta \end{array}$$ (3)

where *θ* are the underlying parameters defining *β*, and *f*(*β*|*θ*) is the probability density of *β*.

As mentioned in the data section, our sample is different from the population in several demographic categories. Our respondents tend to be younger and more educated than the general population, which may result in an upward bias in our WTS estimates.

We estimate a mixed multinomial logit (MMNL) model to account for respondents’ preference heterogeneity by allowing the parameter coefficients to vary across individuals. In addition, we include an alternative specific constant (ASC) in the model so that parameter coefficients vary across choice scenarios, which allows us to differentiate the status quo option and the other two alternatives and accommodate the differences in substitutability between option pairs [30].

We first estimate the MMNL model, allowing the coefficients to be independent (not correlated). The justification of this no-correlation assumption will be further explained in the result section. The coefficient for the number of weeks of staying home is assumed to be log-normally distributed, while the coefficients for all the other attributes are normally distributed. Standard errors are clustered at the individual level. The signs of the coefficient estimates in the MMNL model provide important information on whether a choice attribute improves or reduces individuals’ utility. For example, a positive coefficient for the probability of school opening shows that school opening increases individuals’ utility. On the contrary, a negative coefficient for the number of daily new cases indicates that having a larger number of new cases decreases respondent’s utility and is not preferable.

Following the MMNL model estimation, we then estimate individuals’ WTS home. There are two ways to obtain respondents’ WTS. First, WTS can be calculated using the ratio of coefficients in the utility function as specified in Eq 1 in what is known as preference space estimation [31]. Second, WTS can be estimated directly in the WTS space. The advantage of WTS space estimation is that we can specify the distribution of the WTS directly instead of deriving it indirectly using the distribution of coefficients in the utility function obtained from the preference space. As noted in previous literature, the WTS estimates in the preference space estimation tend to be unrealistically high with less realistic distributions [31, 32]. Another advantage of the estimation in the WTS space is that it accounts for scale heterogeneity among individuals even without a fully correlated model. Hence, we estimate individuals’ WTS home in the WTS space following [31].

In the WTS space estimation, we assume WTS for an increase in the daily number of new cases and unemployment insurance claims to be log-normally distributed with a negative sign, while WTS for schools opening follows a log-normal distribution with a positive sign. We make these log-normal distribution assumptions since WTS home is likely to incur a diminishing marginal return. Therefore, the longer people stay home, the lower their WTS will be. Moreover, we assume the WTS for an increase in the daily number of new cases and unemployment insurance claims have log-normal distributions with negative signs based on the insights we obtained from the preference space estimation. For instance, our estimations in the preference space without imposing directional restrictions on the number of daily cases produce a negative coefficient, which indicates that an increase in the number of daily cases reduces individual’s utility (Table 2). Thus, an increase in daily new cases is considered a disamenity. This result provides us valid reasons to believe that WTS is likely to be negative since people would be willing to stay at home to reduce the increase in new cases. A similar pattern can be observed from the preference space estimation for the unemployment insurance claims attribute. The WTS for mask mandate is assumed to be normally distributed.

**Table 2:** The MMNL results estimated in preference space.

Mean	(1) Full sample	(2) Main sample	(3) × SAH
Alternative specific constant	-2.329*** (0.438)	-2.183*** (0.239)	-1.799*** (0.198)
Number of daily cases	-0.453*** (0.067)	-0.489*** (0.079)	0.296* (0.168)
Weekly unemployment benefit claims	-0.127*** (0.017)	-0.139*** (0.019)	-0.269*** (0.049)
Probability of school opening	0.007*** (0.002)	0.008*** (0.002)	-0.007 (0.005)
Mask wearing mandate = 1	1.356*** (0.096)	1.673*** (0.123)	-0.210 (0.235)
Stay-at-home (weeks)	-3.723*** (0.691)	-3.991*** (0.412)	-18.573*** (4.286)
Number of daily cases × SAH effective			-0.934*** (0.189)
Weekly unemployment benefit claims × SAH effective			0.159*** (0.048)
Probability of school opening × SAH effective			0.016*** (0.005)
Mask wearing mandate = 1 × SAH effective			2.094*** (0.275)
**SD**			
Alternative specific constant	3.914*** (0.334)	2.198*** (0.246)	2.330*** (0.180)
Number of daily cases	0.555*** (0.186)	-0.817*** (0.155)	0.390* (0.217)
Weekly unemployment benefit claims	0.188*** (0.036)	0.233*** (0.027)	0.179*** (0.067)
Probability of school opening	0.012*** (0.004)	0.021*** (0.004)	-0.019*** (0.004)
Mask wearing mandate = 1	1.882*** (0.149)	2.061*** (0.148)	1.586*** (0.230)
Stay-at-home (weeks)	1.032*** (0.329)	-1.647*** (0.128)	-10.380*** (2.351)
Number of daily cases × SAH effective			-0.515** (0.216)
Weekly unemployment benefit claims × SAH effective			0.090 (0.095)
Probability of school opening × SAH effective			0.008 (0.012)
Mask wearing mandate = 1 × SAH effective			1.120*** (0.348)
*N*	13104	12240	13104
LR chi2	386.238	633.342	531.641
Prob >chi2	0.000	0.000	0.000
Log lik.	-3076.578	-2894.033	-3001.180

Standard errors in parentheses.

* *p* < 0.1,

** *p* < 0.05,

*** *p* < 0.01

The table shows the MMNL model estimated in the preference space. Column (1) presents the results for the full sample, including all respondents. Column (2) presents the results for the main sample. We construct the main sample by dropping respondents who always choose the status quo option and do not believe in SAH policy. Column (3) shows the MMNL results when including interactions between choice attributes and a dummy indicating if an individual believes in a SAH order.

The interpretation of our WTS estimate is similar to those of the WTP estimates in a traditional DCE study with a price attribute. Specifically, the estimates indicate how many more or fewer weeks on average respondents are willing to stay home for a unit change in a choice attribute. For example, a negative WTS for weekly unemployment benefit claims shows that individuals are willing to stay home for fewer weeks to prevent the increase in the number of unemployment insurance claims by 100%, or in other words to prevent the number of unemployment insurance claims from growing. Similarly, a positive WTS for schools opening indicates the additional number of weeks individuals are willing to stay at home to improve the possibility of schools opening.

To further investigate the relationship between respondents’ characteristics and their WTS, we first recover the average conditional individual-attribute-specific marginal WTS [33]. We then regress individual’s WTS for each attribute on their socioeconomic and pandemic related characteristics. The estimated results provide insight on heterogeneity in WTS and particularly how individual-specific characteristics affect their WTS.

## Results

### Main MMNL results

The preference space MMNL estimation results are in Table 2. The MMNL method also produces a set of standard deviations that are reported in table. The first column presents the results for the full sample including all respondents in the three states. We have 59 (8.07%) respondents who always choose the status quo option of no SAH orders. These respondents may be protesting the SAH policy, or the complexity of the choice experiment leads to their choice [34–36]. While our survey includes multiple attributes that may make the selection a complex task, we believe the main reason that a few respondents always selected the status quo option is their lack of trust in the SAH policy. First, the number of respondents constantly choosing the status quo option is relatively low compared to other studies, such as [34]. In addition, among those who always choose the status quo, 48 (81.35%) of them also state that they don’t believe in the effectiveness of the SAH policy. As a result, we construct a main sample by dropping respondents who always choose the status quo option of no SAH orders and do not believe that SAH policies are effective in preventing the spread of the virus.

Column (1) and Column (2) compare the MMNL results using the full sample and the main sample separately. In both specifications, all coefficients are highly significant with expected signs. Respondents prefer to have SAH orders with a shorter length. Longer staying home period lowers utility. Not surprisingly, an increase in the number of cases and unemployment insurance claims reduces utility. On the contrary, people gain utility from an increase in the probability of schools opening and a statewide mask mandate. The ratio of the coefficients for the increase in the number of daily new cases and weekly unemployment insurance claims provides essential insight. If we assume that the change in utility with respect to the change in the number of cases and weekly unemployment insurance claims is linear, the ratio of the two coefficients shows us the trade-off between an increase in the number of cases and an increase in the number of weekly unemployment insurance claims.

While controversial like a SAH mandate, it is notable that a statewide mask mandate has an opposite impact on individuals’ utility compared to a SAH order. Specifically, mask-wearing improves utility. This can be potentially attributed to the fact that the inconvenience of wearing masks is relatively low against its extensive benefits, while the cost involved in staying home is much higher. Wearing a mask, like staying home, generates positive externalities. It not only lowers an individual’s probability of getting sick, but also generates external benefits to everyone else by preventing the spread of the virus. These results suggest, in general, mitigation policies such as mask mandates are more favorable than strict suppression policies like lockdown orders. Our findings are consistent with the existing literature findings that there is widespread support for mitigation policies such as mask or face covering mandates [37].

Column (3) shows the MMNL results of an extended model when we include interactions between choice attributes and a dummy variable indicating if a respondent believes in the effectiveness of SAH orders in curbing the spread of COVID-19. The highly significant coefficients (0.05% level) demonstrate that there is a substantial difference in how each attribute affects the respondents’ utility depending on whether they are a believer of the policy or not. The probability of schools opening, and a mask-wearing mandate do not significantly change the utility of non-believers of SAH orders. In contrast, individuals who believe in the SAH orders’ effectiveness lose greater utility from an increase in the number of positive cases, face less utility reduction from an increase in weekly unemployment insurance claims, and gain more utility from an increase in the probability of schools opening and from a mask-wearing mandate than non-believers. The coefficients for the length of a SAH order are negatively significant at the 5% level in all three model specifications. This indicates that staying home is indeed a non-monetary “cost” that reduces respondents’ utility. We also want to acknowledge that though we assume staying home is a cost, being home brings benefits for some individuals, such as more family time, a flexible work schedule, and avoided commuting costs. Considering this, the estimates of WTS for the number of new cases and weekly unemployment insurance claims are likely biased upward. In other words, our estimated WTS might be larger than they are in reality. The negative coefficient can be interpreted as the “net cost” of staying home.

### Willingness-to-stay results

Table 3 presents the mean and median WTS estimates from the WTS space model. All attributes are assumed to be random and independent of each other. Given that we assume the WTS for an increase in daily new cases, unemployment insurance claims, and the probability of schools opening are distributed log-normally, median WTS estimates are more informative than mean WTS. The mask mandate is added as an attribute because it is an important policy tool to curb the pandemic. Proper mask wearing has been shown to affect the number of positive cases. Adding this attribute also improves the flexibility of the choice alternatives and ensures the choice scenarios are realistic. However, the mask mandate’s WTS coefficient should not be interpreted as the number of weeks individuals are willing to stay at home to impose a mask mandate given that a mask mandate is not directly affected by individuals’ staying home choices. Therefore, we focus our discussion mainly on median WTS for an increase in daily new cases, unemployment insurance claims, and the probability of schools opening. We also present the WTS results using the full sample in S1 Table in S1 File.

**Table 3:** Estimated WTS for different attributes in the WTS space.

	Mean	Median	SD
Number of daily cases	-21.624** (10.117)	-5.488* (3.110)	82.416 (53.888)
Weekly unemployment benefit claims	-4.667** (2.156)	-1.997** (0.996)	9.858* (5.252)
Probability of schools opening	0.208** (0.104)	0.191* (0.101)	0.090 (0.100)
Mask wearing mandate = 1	45.671** (20.469)	45.671** (20.469)	56.901** (25.410)
Alternative specific constant	-61.166** (25.728)	-61.166** (25.728)	-65.801** (29.647)
*N*	12,240	12,240	12,240

Standard errors in parentheses

* *p* < 0.1,

** *p* < 0.05,

*** *p* < 0.01

This table shows the estimated mean, median, and standard deviation of WTS for four choice attributes. The model is estimated in WTS space. WTS of attribute “Mask wearing mandate” is assumed to be normally distributed, while the WTS for the other attributes are assumed to be log-normally distributed. The WTS for alternative specific constant is included and assumed to be normally distributed.

Consistent with previous literature [38], we confirm individuals’ strong support for the SAH orders. Our results suggest that respondents are willing to stay home for additional five and half weeks to lower the increase of new cases by 100%. Their WTS to reduce the increase in the unemployment insurance claims by 100% is approximately two weeks, which is much lower than the WTS for case reduction. The fact that individuals are less willing to stay home to reduce unemployment insurance claims than to prevent disease infection indicates that they recognize the trade-off between infection prevention and economic development slowdown caused by SAH orders. While a SAH mandate generates benefits by reducing the spread of the virus, it seizes businesses and reduces economic activity, which leads to job losses and an increase in the number of weekly unemployment insurance claims, resulting in large economic cost [39] and mental health costs [6, 7].

Individuals’ WTS to increase the probability of schools opening in the fall is relatively large. Specifically, respondents are willing to stay home for about nine and half weeks to increase the probability of schools opening by 50%. Given that schools opening may affect the number of positive cases when students gather, this WTS estimate highlights the importance of schools opening to the general public.

We note that our estimated WTS coefficients are based on the uncorrelated model. To justify this assumption and properly specify the correlation structure between the choice attributes, we compare the results of the uncorrelated and the fully correlated model. We find that the uncorrelated model has a favorable Bayesian Information Criterion (BIC). In addition, we test the correlation between the choice attributes in the uncorrelated model. As shown in Table 4, the correlations between the four choice attributes are low. We have also tested the correlation in a fully correlated model as a robustness check, and the results are comparable to these in the uncorrelated model. We, therefore, decide to estimate individuals’ WTS in the WTS space with an uncorrelated MMNL model. As noted earlier, the uncorrelated model accounts for scale heterogeneity. The scale parameter captures all sources of variation that exist for the coefficients in the utility function even when only scale heterogeneity is controlled. Hence, our uncorrelated model captures other variations among utility coefficients as well.

**Table 4:** Correlation between WTS for the main attributes.

	Number of daily cases
Weekly unemployment benefit claims	-0.057
Probability of schools opening	-0.007
Mask wearing mandate = 1	0.092
*N*	680

This table shows the correlation between the choice attributes in the uncorrelated model estimated in the WTS space. We use the main WTS-space specification (Table 3) to recover conditional individual-specific WTS.

Absent a SAH mandate, the unmitigated spread of COVID-19 can also reduce economic activity due to the reduced human interactions stemming from individuals’ defensive behavior [12]. Thus, while individuals perceive a trade-off between costs and benefits of a SAH mandate at the levels observed in August and exhibit certain preference towards the SAH order, this outcome does not necessarily hold across different stages of the pandemic when the transmission rates and economic conditions vary.

### Heterogeneity in WTS

We may expect individuals to display heterogeneous WTS. Individuals experience different costs and benefits from a statewide SAH policy. Those who are more vulnerable to the disease, like the old, may be more willing to advocate for such mandates than the young. On the other hand, those who rely on jobs requiring human interactions may be less willing to support a stay-at-home policy.

The high standard deviations of the WTS estimates as shown in Table 3 provides support for our hypothesis that there is a significant amount of heterogeneity in individuals’ preferences and WTS home.

Table 5 presents the estimation results when we regress respondents’ WTS for each attribute on their demographics like age, gender, income, and pandemic-related characteristics such as whether they can work from home or whether they are health or essential workers.

**Table 5:** Heterogeneity in WTS for different attributes.

	Number of daily case	Weekly unemployment benefit claims	Probablity schools opening
Believe SAH effective	-6.605 (4.132)	2.087*** (0.595)	0.002 (0.001)
Work from home	-3.571 (3.165)	0.765* (0.456)	0.001 (0.001)
Active employment	0.417 (3.317)	0.126 (0.478)	-0.001 (0.001)
Senior (above 65)	6.117* (3.376)	-0.135 (0.486)	-0.002* (0.001)
Bachelor degree and above	-1.044 (3.017)	0.158 (0.434)	0.001 (0.001)
Believe free-riding exits^a^	-5.934 (4.138)	0.386 (0.596)	-0.001 (0.001)
Not envious^b^	-1.497 (3.229)	-0.600 (0.465)	0.001 (0.001)
Envious^b^	0.615 (3.003)	0.685 (0.432)	-0.001 (0.001)
Republican	0.608 (3.316)	0.012 (0.478)	0.001 (0.001)
Received unemployment insurance	-6.128* (3.691)	0.469 (0.532)	0.000 (0.001)
Health worker	-4.998 (3.174)	0.090 (0.457)	-0.001 (0.001)
Essential worker	0.038 (3.113)	0.060 (0.448)	0.001 (0.001)
Experienced wage cut	4.000 (3.288)	-0.511 (0.474)	0.000 (0.001)
Female	0.764 (2.918)	-0.397 (0.420)	-0.000 (0.001)
Income >100K	5.251 (3.550)	-0.460 (0.511)	-0.001 (0.001)
Conservative for economic issues^c^	1.917 (3.363)	-0.412 (0.484)	0.000 (0.001)
Constant	-13.539** (5.490)	-6.361*** (0.791)	0.207*** (0.002)
*N*	673	673	673

Standard errors in parentheses

* *p* < 0.1,

** *p* < 0.05,

*** *p* < 0.01

This table shows the OLS regression results when we regress individual-attribute-specific WTS on individual characteristics.

^*a*^We ask respondents what percentage of people in their community they believe are not wearing masks to measure if they believe there exists free-riding.

^*b*^We ask respondents about how the wealth of others impacts their own happiness. The two question reads as: “Using the provided scale (1 “strongly disagree” to 5 “strongly agree”), please indicate to what extent you agree or disagree with the following statement: “Regardless of how much money I have, I am concerned that there are people who have more (less) money than me”. A selection of 5 or 4 in these two questions would indicate that the respondent identifies as being envious (not envious) of the wealth of others.

^*c*^We ask respondents their political ideology for economic issues. Choices include slightly conservative, middle of the road, slightly liberal, and liberal. To preserve the degrees of freedom, we have redefined respondents as conservative for economics issues if he/she chooses conservative and slightly conservative in this question. If respondents claim that their views on economic issues are conservative or slightly conservative, the variable conservative for economic issues equals 1 and 0 otherwise.

Results are generally consistent with our expectations and follow intuition. Seniors who are 65 or older have substantially higher WTS, over 6 weeks, to a lower number of cases than younger individuals. This finding also confirms our hypothesis that older respondents are warier about the disease. In contrast, seniors have slightly lower WTS to increase the probability of schools opening. Additionally, individuals who have applied for unemployment insurance claims are willing to stay home 6 fewer weeks to bring down positive cases than those who do not. Given that staying home results in stagnation in the labor market, this result is not surprising. We find that respondents who can work from home are willing to stay home for 0.8 additional weeks to increase the number of unemployment insurance claims than those who cannot. In other words, their WTS to reduce unemployment is lower than their counterparts. A possible explanation is that people who have the option to work from home are not concerned about losing their jobs, leading to lower WTS for reducing unemployment. Together, these results validate our hypothesis that individuals bear different costs and benefits from the SAH policy, and as a result, have a heterogeneous WTS to reduce the spread of the virus.

## Discussion

This paper empirically quantifies individuals’ WTS home during the COVID-19 pandemic by conducting a stated-preference DCE survey and further explored factors driving the heterogeneity in individuals’ WTS. Our findings can provide critical information needed for effective and efficient policy responses during the COVID-19 pandemic and future pandemics.

With winter approaching and individuals gathering and spending more time indoors, countries worldwide are experiencing another COVID-19 outbreak with surging cases. We find that staying home results is a net cost to individuals and lowers their utility. However, the magnitude of respondents’ WTS to reduce the number of cases and increase the probability of schools opening is still quite large even when the net cost is considered. Specifically, we find that by keeping the number of unemployment benefits claims fixed, individuals are willing to stay home for approximately an additional five weeks to reduce the number of positive COVID-19 cases by 100%. However, if a SAH order would increase the number of unemployment benefits claims, our results suggest that people do consider the rise in unemployment and shorten their WTS length. [40] show that individuals are more willing to obey SAH policies if the length of a SAH order is no longer than they expected. Our findings provide insight into the potential length of SAH policies if the re-implementation of lockdown is considered.

There are continuing heated debates and deeply divided views on the mask mandate across the country, though mask-wearing has been recognized as one of the most effective tools to curb the pandemic in the public health community. Overall, we present evidence showing that the average respondent gains utility from mask and face-covering mandates. Policymakers may consider leveraging such support to utilize mask mandates as an effective preventative measure limiting the further spread of the virus.

In addition, we find that several pandemic related characteristics, including age, ability to work from home, and employment status are factors determining individuals’ heterogeneous WTS. Targeted policies that differentiate the population by age and risk groups have been found to outperform uniform lockdown policies in theory. Based on the results of this study, specific suggestions on how potential targeted policies could be implemented are offered.

## Conclusion

Our study values attributes related to the pandemic (e.g. case counts) using the amount of time individuals are willing to stay indoors, rather than a dollar amount. We survey individuals across California, Illinois, and Georgia, and use a DCE to elicit WTS measures. We then use discrete choice modeling techniques to calculate WTS for our study region, and find evidence that there is heterogeneity in WTS depending on a variety of demographic variables which are captured in our survey.

This study makes several major contributions. First, to the best of our knowledge, this is the first study that explicitly measures individuals’ WTS home during the COVID-19 pandemic in three major states in the United States. In particular, we conduct a DCE survey to investigate how individuals perceive trade-offs between the benefits such as disease prevention and the costs including both economic and mental health costs of a SAH order. Previous studies have mainly focused on the trade-offs from a social planner’s point of view and suggest that SAH policies, especially targeted ones based on age and other risk factors can be effective [8–15]. However, successful policy implementation depends on individuals’ preferences and WTS home. Existing research has investigated the extensive margin of individuals’ preferences towards SAH orders; that is, whether individuals are willing to comply with mandatory SAH orders. Our analysis contributes to the literature by measuring individuals’ WTS at the intensive margin—how long they are willing to stay home.

While [16] have evaluated individuals’ willingness-to-accept (WTA) to stay home in Sweden, we examine people’s WTS in the United States. We show that individuals are willing to stay home for additional five and half weeks to lower the increase in new cases by 100%. Previous literature indicates that individuals are less willing to obey SAH policies when presented with a longer SAH length than expected [40]. Our findings provide suggestive evidence that the general public’s support for SAH policies is still strong as the pandemic progresses.

Second, our survey design limits the impact of free-riding when quantifying individuals’ WTS. In our DCE survey, instead of asking respondents how many weeks they are willing to stay home directly as in existing surveys (e.g. [41]), we use “the length of a SAH order” to measure their WTS. We also reminded respondents multiple times that the SAH mandate is strictly imposed by the government and will severely limit their daily activities if in place. Staying home lowers the possibility of getting sick for not only the person himself/herself but also everyone else in the community. These unintentional and uncompensated benefits are identified as positive externalities in economics. Economic theory predicts that free-riding is likely to occur with public goods like staying-at-home behavior when the benefits are both non-excludable and non-rival; that is, nobody can be excluded from receiving the benefit and one person’s consumption does not reduce others’ utility [42]. When a strict SAH policy with proper enforcement is implemented, no matter how individuals perceive the external benefits of staying home, they need to comply with the regulation. Our survey design therefore provides an estimate closer to the socially optimal level.

Third, we contribute to the non-market valuation literature. Our evaluation of individuals’ opportunity cost to staying home is similar to the travel cost method in revealed preference valuation literature, which considers time spent on a trip as part of the travel cost to evaluate the value of non-marketed environmental goods [43]. In addition, recent stated preference studies have adopted non-monetary payment modes to elicit respondents’ preferences for non-market goods and services. For example [44], have estimated respondents’ willingness to volunteer their time in stormwater management. [45] has investigated individuals’ willingness to work. We further expand the literature by quantifying individuals’ WTS home during an unprecedented pandemic.

We recognize one limitation in our study regarding the magnitudes of our estimates. Even though we have reminded the strictness of a SAH order in our survey, respondents might choose longer lengths of SAH orders than they would have accepted in reality since the SAH orders are not easily enforceable. In other words, this brings a potential upward bias into our estimates.

There are several avenues to expand the work and advance our understanding of people’s WTS home during pandemics. First, we fill in the gap in the literature on measuring individuals’ stated WTS, which can be interpreted as individuals’ stated willingness to comply with a new SAH order. Since we include different lengths of SAH as attributes, we assume individuals are willing to comply if the new SAH order is within their WTS. However, non-compliance can potentially bias our estimates towards longer WTS. Future research can use revealed preference data to measure individuals’ actual compliance. Second, our choice experiment design focuses on state-level SAH policies, which limits our ability to address the potential conflicts between state and local variations in a SAH order. Past literature has shown that regulating agency is an important attribute affecting individuals’ choices [46]. In particular, the authorities issuing the SAH home order may affect people’s compliance [47]. Future research can further explore the relationship between the types of authority and people’s WTS at home. Third, a retrospective survey at the end of the pandemic evaluating what individuals believe would have been optimal at the beginning of the pandemic may inform policy given that individuals become more informed over time. In addition, future studies may examine more nuanced SAH policies, for example, with varying degrees and types of closures (e.g., park closures versus bar closures). A study that examines methods and levels of enforcing SAH orders and mask-wearing mandates may also add additional insight into the policy implementation.

## Supporting information

S1 File (DOCX)
