# Supplementary material for: Stay-at-home orders and the willingness to stay home during the COVID-19 pandemic: A stated-preference discrete choice experiment
Source: PLoS One. 2021 Jul 1;16(7):e0253910. doi: 10.1371/journal.pone.0253910 (PMC8248689; doi:10.1371/journal.pone.0253910)

**Supporting Information for Stay-at-Home Orders and the Willingness to Stay Home During the COVID-19 Pandemic: A Stated-Preference Discrete Choice Experiment**

**S1. Full Survey**

Below we present the full survey given to our respondents. As mentioned in the paper, we have three blocks of six questions within each block for California, Illinois, and Georgia. Here we only show six choice cards, which is the number of choice cards that was shown to each respondent, to avoid repetition and reduce the size of the appendix.

**Screening**

Age?

Under 18 (0)

- 1. (1)

25-34 (2)

35-44 (3)

45-54 (4)

55-64 (5)

65+ (6)

Which state do you currently reside in?

California (1)

Georgia (2)

Illinois (3)

Other (4)

What is your current zip code?

________________________________________________________________

**Introduction**
  
You are invited to participate in a survey investigating how long people are willing to stay at home to prevent the spread of the Coronavirus (COVID-19). The survey results will improve understanding of how factors such as unemployment and the spread of COVID-19 impact people’s preferences for staying at home.
Participation in this study is voluntary, and you can withdraw anytime. There are no foreseeable risks if you choose to continue.  Your responses will be recorded anonymously. No identifying personal information will be collected.  **It should take you approximately 15 minutes to complete the survey diligently.**
  
The information collected during the study could be used for future research without additional informed consent. If you have any questions or concerns about your rights in this research study, please contact the Clemson University Office of Research Compliance (ORC) at 864-656-0636 or irb@clemson.edu.  If you are outside of the Upstate South Carolina area, please use the ORC’s toll-free number, 866-297-3071. The investigators, Dr. Mani Rouhi Rad. (rrad@clemson.edu), or Dr. Dede Long (Dede.Long@csulb.edu) can be contacted for study-specific questions. If you agree to participate in this study, please continue.

**Note:**Because the goal of this research is to understand individual preferences, your responses matter!  The results of this study could influence policies and practices related to the Coronavirus (COVID-19) pandemic.  To ensure data quality, it is important that you **carefully read all the information provided, and that you thoughtfully give your best answer to each question in the survey.**

**Consent**

Do you commit to carefully reading and providing your thoughtful and honest answers to the questions in this survey?

I will read carefully and provide my best answers (1)

I will not read carefully and provide my best answers (0)

I can't promise either way (0)

**Background Information**

The novel Coronavirus has caused a recent outbreak of the respiratory illness COVID-19.  **It is a consensus among scientists and researchers that social and physical distancing is an important containment measure of COVID-19.**

Many suggest that implementing a stay-at-home/shelter-in-place order is **necessary** to reduce the spread and severity of COVID-19.  However, stay-at-home orders have both economic costs (i.e., unemployment) and psychological costs (i.e., mental health issues due to isolation and the lack of human interaction).

Effective prevention or treatment, such as a vaccine against the COVID-19 is therefore a top priority.  Most experts think that a vaccine is likely to become available by mid-2021, approximately 12 months from now.  Before a vaccine is available, reopening may increase people’s social interactions and the possibility of virus transmission.

The previous stay-at-home/shelter-in-place order has been lifted gradually in most states, allowing businesses such as hair salons to re-open.  However, a surge in cases has been observed in many reopened states (e.g. California), leading some to pause the reopening.  A new statewide stay-at-home/shelter-in-place is considered as a tool to prevent the further spread of the Coronavirus.  The next page provides the survey instructions.  Please read the descriptions carefully before starting the survey.   

**Survey Instructions**
 
We are interested in understanding how many additional weeks people are willing to stay at home to prevent the spread of coronavirus before vaccines become available. The survey has two parts.

In the **first part** of the survey, you will be asked **six** choice questions.  In each of those questions, we will ask you to choose between two possible scenarios depending on the number of weeks you choose to stay at home, compared to a status quo situation.  In this experiment, choosing the status quo situation means you prefer the government to reopen the state without staying at home for any weeks.

In the **second part**, we ask you some short questions regarding your demographic background.  Answers to these questions are important for our understanding of what factors may affect your choices regarding whether or not to stay additional weeks at home during the pandemic. **The survey results could affect decisions made by relevant actors regarding public policy to prevent the spread of the coronavirus and/or address its economic and psychological impact.**

Status quo scenario without a new stay-at-home order:

Without any new stay-at-home policies, your state is currently partially open, which allows businesses such as:

1. Retail stores and salons to reopen with capacity and other limits and safety precautions;
2. Restaurants to reopen outdoor/indoor seating with social distancing requirements still in place.

This is called the status quo scenario in the survey.

Fixed features when a new stay-at-home order is implemented:

If a new stay-at-home policy is implemented, for the purposes of this survey you should assume that every possible stay-at-home scenario has the following features:

1. Essential businesses (e.g., healthcare operations, grocery stores, etc.) are still open.
2. Non-essential businesses (e.g., gyms, theaters, etc.) are closed.  Residents are expected to stay in their houses as much as possible.  They may leave home to:

a. Take care of essential needs such as getting food and medicine, going to the doctor, exercising, and caring for your loved ones and pets;

b. Work and travel if it is considered essential, handle basic business needs such as maintaining the business’s basic operational needs;

1. Grocery prices stay the same; stores have adequate supplies of household essentials such as toilet paper and hand sanitizer.

**Please remember that the stay-at-home order is STRICT. Your daily activities will be extremely restricted.**

CALIFORNIA **Variable features of stay-at-home scenarios**: If a new stay-at-home policy is implemented, depending on how long people stay at home, the spread and severity of the coronavirus illness (COVID-19) and its potential economic impact may vary. The variable features of staying at home scenarios described as follows are of interest in this survey.  Please read them carefully before answering the survey questions.

| **Attribute and Description** | **Attribute Levels** |
| --- | --- |
| Length of a state-wide stay-at-home order | 4 weeks  6 weeks  8 weeks  10 weeks |
| Mask Wearing Policy:  Whether the public is required to wear a mask or face covering when they leave home and are unable to socially distance (6 feet) over the next **TEN weeks (2.5 months)**. | Wearing a mask or face covering is required.  Wearing a mask or face covering is not required. |
| Increase in newly confirmed COVID-19 cases (daily):  Increase in newly confirmed COVID-19 cases **every day** in your state **TEN weeks (2.5 months) from today**. A higher number means more people are infected by the coronavirus every day.  Note: The number of newly confirmed COVID-19 cases in California is 5,920 as of August 20^th^. | 0.5 times (50%) increase from today  1 time (100%) increase from today  1.5 times (150%) increase from today  2 times (200%) increase from today |
| Increase in weekly *initial* unemployment insurance claims:  Increase in weekly emerging unemployment insurance claims in your state **TEN weeks (2.5 months) from** **today**. A higher number means more people have filed unemployment insurance claims.  Note: The *Unemployment Insurance* program pays benefits to workers who have lost their jobs and meet the program's eligibility requirements.  The number of pre-pandemic (03/14/2020) weekly initial unemployment insurance claims is 57,606 in California. | 2 times the pre-pandemic level  4 times the pre-pandemic level  6 times the pre-pandemic level  8 times the pre-pandemic level |
| School Opening Possibility:  Probability of schools being open **FOUR weeks from** **today**. | 20% of chance that schools are open  40% of chance that schools are open  60% of chance that schools are open  80% of chance that schools are open |

ILLINOIS **Variable features of stay-at-home scenarios**: If a new stay-at-home policy is implemented, depending on how long people stay at home, the spread and severity of the coronavirus illness (COVID-19) and its potential economic impact may vary. The variable features of staying at home scenarios described as follows are of interest in this survey.  Please read them carefully before answering the survey questions.

| **Attribute and Description** | **Attribute Levels** |
| --- | --- |
| Length of a state-wide stay-at-home order | 4 weeks  6 weeks  8 weeks  10 weeks |
| Mask Wearing Policy:  Whether the public is required to wear a mask or face covering when they leave home and are unable to socially distance (6 feet) over the next **TEN weeks (2.5 months)**. | Wearing a mask or face covering is required.  Wearing a mask or face covering is not required. |
| Increase in newly confirmed COVID-19 cases (daily):  Increase in newly confirmed COVID-19 cases **every day** in your state **TEN weeks (2.5 months) from today**. A higher number means more people are infected by the coronavirus every day.  Note: The number of newly confirmed COVID-19 cases in Illinois is 1,832 as of August 20^th^. | 0.5 times (50%) increase from today  1 time (100%) increase from today  1.5 times (150%) increase from today  2 times (200%) increase from today |
| Increase in weekly *initial* unemployment insurance claims:  Increase in weekly emerging unemployment insurance claims in your state **TEN weeks (2.5 months) from** **today**. A higher number means more people have filed unemployment insurance claims.  Note: The *Unemployment Insurance* program pays benefits to workers who have lost their jobs and meet the program's eligibility requirements.  The number of pre-pandemic (03/14/2020) weekly initial unemployment insurance claims is 11,305 in Illinois. | 2 times the pre-pandemic level  4 times the pre-pandemic level  6 times the pre-pandemic level  8 times the pre-pandemic level |
| School Opening Possibility:  Probability of schools being open **FOUR weeks from** **today**. | 20% of chance that schools are open  40% of chance that schools are open  60% of chance that schools are open  80% of chance that schools are open |

GEORGIA **Variable features of stay-at-home scenarios**: If a new stay-at-home policy is implemented, depending on how long people stay at home, the spread and severity of the coronavirus illness (COVID-19) and its potential economic impact may vary. The variable features of staying at home scenarios described as follows are of interest in this survey.  Please read them carefully before answering the survey questions.

| **Attribute and Description** | **Attribute Levels** |
| --- | --- |
| Length of a state-wide stay-at-home order | 4 weeks  6 weeks  8 weeks  10 weeks |
| Mask Wearing Policy:  Whether the public is required to wear a mask or face covering when they leave home and are unable to socially distance (6 feet) over the next **TEN weeks (2.5 months)**. | Wearing a mask or face covering is required.  Wearing a mask or face covering is not required. |
| Increase in newly confirmed COVID-19 cases (daily):  Increase in newly confirmed COVID-19 cases **every day** in your state **TEN weeks (2.5 months) from today**. A higher number means more people are infected by the coronavirus every day.  Note: The number of newly confirmed COVID-19 cases in Georgia is 2,812 as of August 20^th^. | 0.5 times (50%) increase from today  1 time (100%) increase from today  1.5 times (150%) increase from today  2 times (200%) increase from today |
| Increase in weekly *initial* unemployment insurance claims:  Increase in weekly emerging unemployment insurance claims in your state **TEN weeks (2.5 months) from** **today**. A higher number means more people have filed unemployment insurance claims.  Note: The *Unemployment Insurance* program pays benefits to workers who have lost their jobs and meet the program's eligibility requirements.  The number of pre-pandemic (03/14/2020) weekly initial unemployment insurance claims is 5,445 in Georgia. | 2 times the pre-pandemic level  4 times the pre-pandemic level  6 times the pre-pandemic level  8 times the pre-pandemic level |
| School Opening Possibility:  Probability of schools being open **FOUR weeks from** **today**. | 20% of chance that schools are open  40% of chance that schools are open  60% of chance that schools are open  80% of chance that schools are open |

This question is only an example. Your response here will not be recorded.
Given the fixed and variable features that are previously presented, please answer the following choice question.  
Note: you will **NOT** be able to go back and change your answers for the choice questions, so please think carefully. 

Again, please remember that the stay-at-home order is STRICT. Your daily activities will be extremely restricted.

 
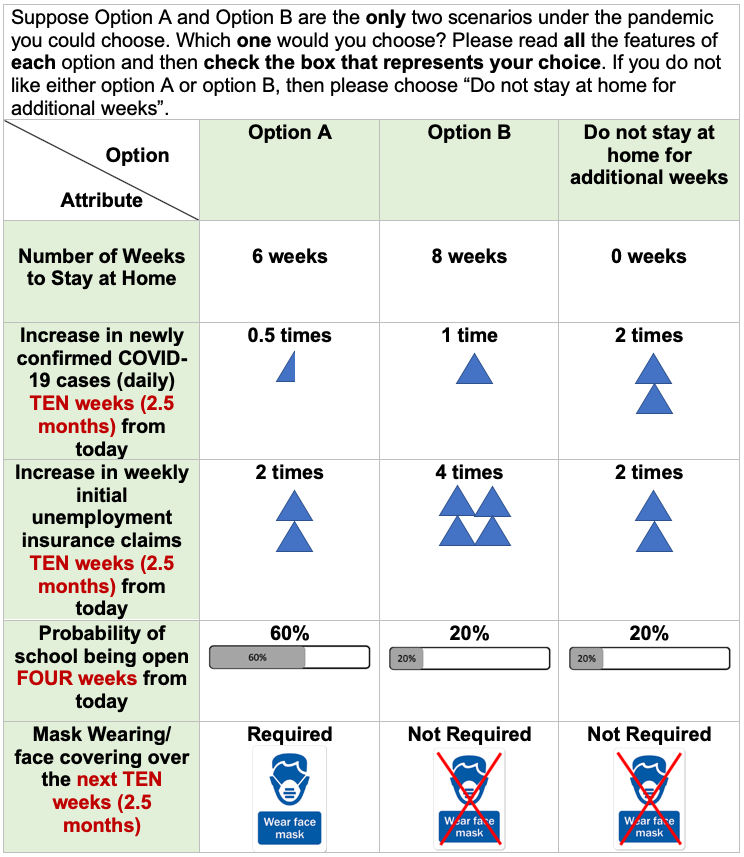


Option A (1)

Option B (2)

Status Quo - Do not stay at home (3)

**Choice Question 1**


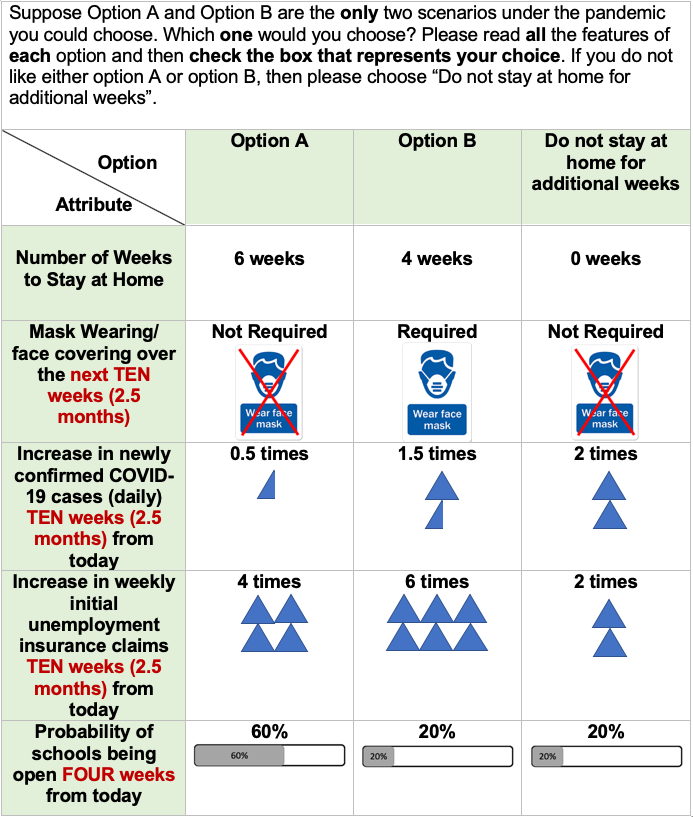


Option A (1)

Option B (2)

Status Quo - Do not stay at home (3)

**Choice Question 2**


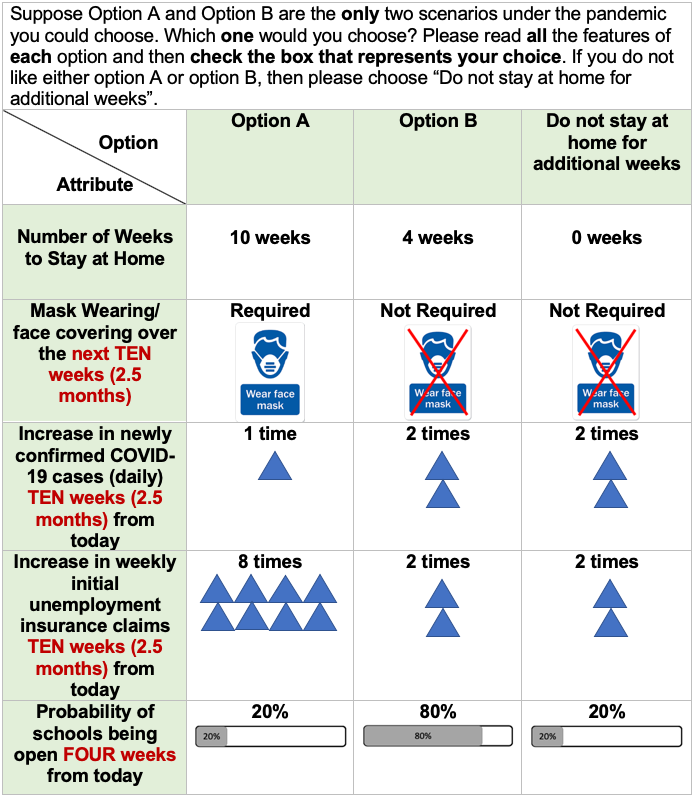


Option A (1)

Option B (2)

Status Quo - Do not stay at home (3)

**Choice Question 3**


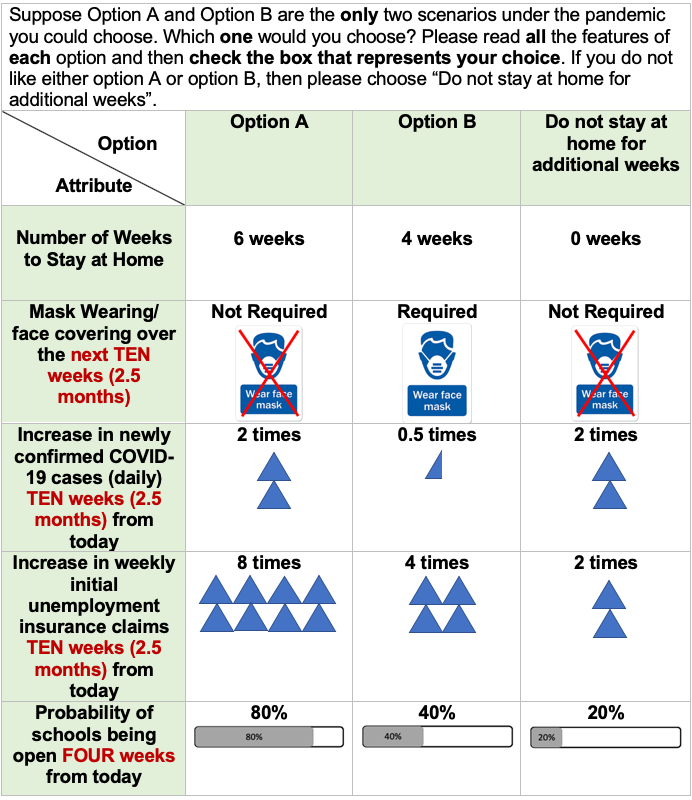


Option A (1)

Option B (2)

Status Quo - Do not stay at home (3)

**Choice Question 4**


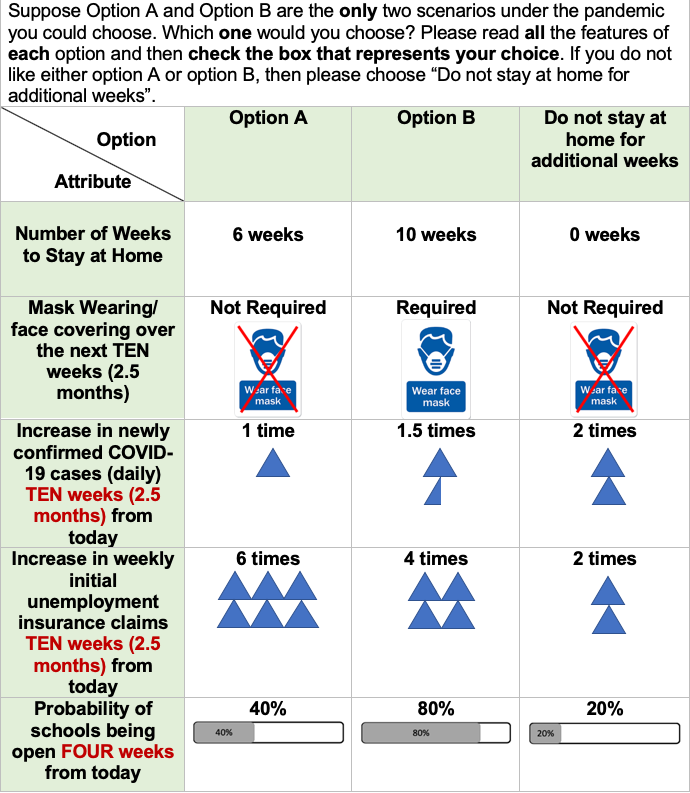


Option A (1)

Option B (2)

Status Quo - Do not stay at home (3)

**Choice Question 5**


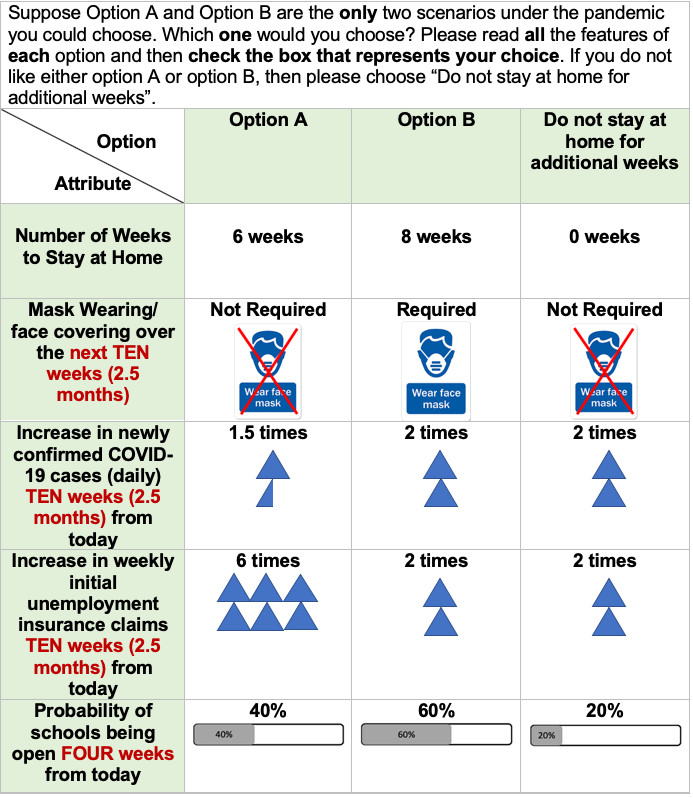


Option A (1)

Option B (2)

Status Quo - Do not stay at home (3)

**Choice Question 6**


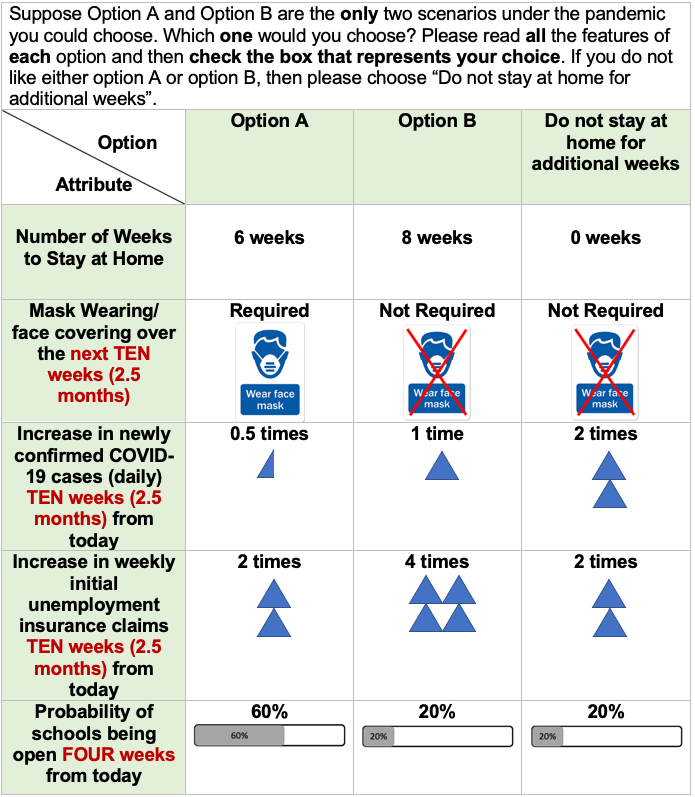


Option A (1)

Option B (2)

Status Quo - Do not stay at home (3)

On a scale of 1 to 5, where 1 indicates “Not certain at all” and 5 indicates “Very certain”, could you indicate the degree of certainty about your responses to the questions above?

1 - Not at all (1)

2 (2)

3 (3)

4 (4)

5 - Very Much (5)

To what extent do you believe that your answers in this survey will be taken into account by policy makers and any other actors of COVID-19 health crisis as well as to the wider general public?

1 - Not at all (1)

2 (2)

3 (3)

4 (4)

5 - Very Much (5)

Do you think the stay-at-home order is an effective policy to slow the spread of the coronavirus?

Yes (1)

No (5)

Using the provided scale, please indicate to what extent you agree or disagree with the following statement: “*Regardless of how much money I have, I am concerned that there are people who have less money than me*”.

1 - Strongly Disagree (1)

2 (2)

3 (3)

4 (4)

5 - Strongly Agree (7)

Using the provided scale, please indicate to what extent you agree or disagree with the following statement: “*Regardless of how much money I have, I am concerned that there are people who have more money than me*”.

1 - Strongly Disagree (1)

2 (2)

3 (3)

4 (4)

5 - Strongly Agree (7)

Do you have family members who are health workers?

Yes (1)

No (5)

Are you or any of your family members essential workers?

Yes (1)

No (2)

Have you experienced a wage cut/furlough because of COVID-19?

Yes (1)

No (2)

Is work-from-home currently an option for you to do your job?

Yes (1)

No (2)

Did you apply for unemployment benefit?

Yes (1)

No (2)

What job sector(s) are you working in?

Mining (1)

Construction (4)

Manufacturing (2)

Utilities (3)

Wholesale trade (5)

Retail trade (6)

Transportation and warehousing (7)

Information (8)

Financial activities (9)

Professional and business services (10)

Educational services (11)

Health care and social assistance (12)

Leisure and hospitality (13)

Other services (14)

Federal government (15)

State and local government (16)

Agriculture, forestry, fishing, and hunting (17)

Agriculture self-employed (20)

Nonagriculture self-employed (19)

To what extent do you believe that the majority of people in your community wear masks or face coverings when they go out?

1 - Not at all (1)

2 (2)

3 (3)

4 (4)

5 - Very Much (5)

Gender

Male (0)

Female (1)

Other (2)

Which of the following options best describes your ethnicity?

White (1)

Black or African-American (2)

Hispanic (3)

American Indian or Alaska Native (4)

Asian (5)

Native Hawaiin or Pacific Islander (6)

Other (7)

Marital status?

Single (1)

Married (2)

Divorced (3)

Widow(er) (4)

Q126 Which year were you born?

________________________________________________________________

Please indicate the highest level of education that you have completed

Some high school (1)

GED/High school diploma (2)

Some college (3)

Associate's degree (4)

Bachelor's degree (5)

Graduate degree (6)

How many people, including you, live in your household?

________________________________________________________________

How many children under 18 live in your current household?

________________________________________________________________

How many seniors above 65 live in your current household?

________________________________________________________________

In politics today, do you consider yourself a Republican, a Democrat, an Independent, or something else?

Republican (1)

Democrat (2)

Independent (3)

Something else (4)

How would you describe your political ideology in terms of social issues, i.e., abortion?

Liberal (2)

Slightly liberal (3)

Middle of the road (4)

Slightly conservative (5)

Conservative (6)

How would you describe your political ideology in terms of economic issues, i.e., tax rates?

Liberal (2)

Slightly liberal (3)

Middle of the road (4)

Slightly conservative (5)

Conservative (6)

What is your annual household income before taxes?

Less than $25,000 (1)

$25,000 to $49,999 (2)

$50,000 to $74,999 (3)

$75,000 to $99,999 (4)

$100,000 to $149,999 (5)

$150,000 to $199,999 (6)

$200,000 or more (7)

Check all of the following categories that describe you

Student - full time (1)

Student - part time (7)

Recent graduate (8)

Employed - full time (13)

Employed - part time (14)

Self-employed (15)

Retired (9)

Not employed (10)

Other (12)

Over the last 2 weeks, how often have you been bothered by problems such as 
1) Feeling nervous, anxious or on edge
2) Worrying too much about different things
3) Trouble relaxing
4) Becoming easily annoyed or irritable
5) Feeling afraid as if something awful might happen

Not at all (1)

Several days (2)

Over half of the days (3)

Nearly every day (4)

If you have been bothered by the problems in the previous question, how difficult have these made it for you to do your work, take care of things at home, or get along with other people?

Not difficult at all (1)

Somewhat difficult (2)

Very difficult (3)

Extremely difficult (4)

S1 Table: WTS space estimation results using full sample


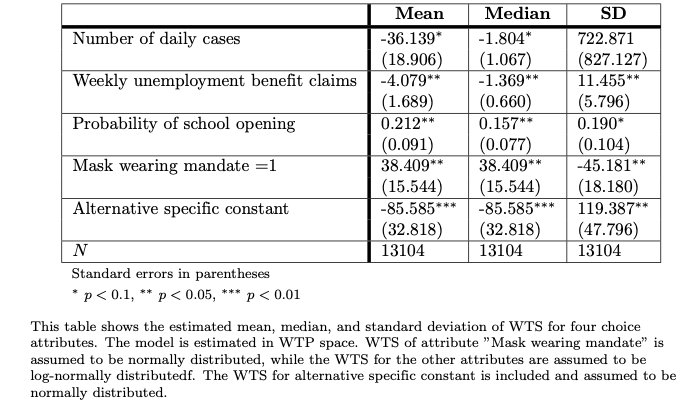

Supplement: S1 File — (DOCX) [file pone.0253910.s001.docx]
